# Receptor-Interacting Protein Kinase 3 Inhibition Prevents Cadmium-Mediated Macrophage Polarization and Subsequent Atherosclerosis *via* Maintaining Mitochondrial Homeostasis

**DOI:** 10.3389/fcvm.2021.737652

**Published:** 2021-11-08

**Authors:** Jiexin Zhang, Weijing Feng, Minghui Li, Peier Chen, Xiaodong Ning, Caiwen Ou, Minsheng Chen

**Affiliations:** ^1^Department of Cardiology, Laboratory of Heart Center, Zhujiang Hospital, Southern Medical University, Guangzhou, China; ^2^Guangdong Provincial Key Laboratory of Shock and Microcirculation, Guangzhou, China; ^3^Department of Cardiology, State Key Laboratory of Organ Failure Research, Nanfang Hospital, Southern Medical University, Guangzhou, China

**Keywords:** cadmium (Cd), atherosclerosis (AS), RIPK3, macrophage polarization, mitochondrial homeostasis

## Abstract

Chronic cadmium (Cd) exposure contributes to the progression of cardiovascular disease (CVD), especially atherosclerosis (AS), but the underlying mechanism is unclear. Since mitochondrial homeostasis is emerging as a core player in the development of CVD, it might serve as a potential mechanism linking Cd exposure and AS. In this study, we aimed to investigate Cd-mediated AS through macrophage polarization and know the mechanisms of Cd-caused mitochondrial homeostasis imbalance. *In vitro*, flow cytometry shows that Cd exposure promotes M1-type polarization of macrophages, manifested as the increasing expressions of nuclear Factor kappa-light-chain-enhancer of activated B (NF-kB) and NLR family pyrin domain containing 3 (NLRP3). Mitochondrial homeostasis tests revealed that decreasing mitochondrial membrane potential and mitophage, increasing the mitochondrial superoxide (mROS), and mitochondrial fission are involved in the Cd-induced macrophage polarization. The upregulated expressions of receptor-interacting protein kinase 3 (RIPK3) and pseudokinase-mixed lineage kinase domain-like protein (p-MLKL) were observed. Knocking out RIPK3, followed by decreasing the expression of p-MLKL, improves the mitochondrial homeostasis imbalance which effectively reverses macrophage polarization. *In vivo*, the oil red O staining showed that Cd with higher blood significantly aggravates AS. Besides, M1-type polarization of macrophages and mitochondrial homeostasis imbalance were observed in the aortic roots of the mice through immunofluorescence and western blot. Knocking out RIPK3 restored the changes above. Finally, the administered N-acetyl cysteine (NAC) or mitochondrial division inhibitor-1 (Mdivi-1), which decreased the mROS or mitochondrial fission, inhibited the expressions of RIPK3 and p-MLKL, attenuating AS and macrophage M1-type polarization in the Cd-treated group. Consequently, the Cd exposure activated the RIPK3 pathway and impaired the mitochondrial homeostasis, resulting in pro-inflammatory macrophage polarization and subsequent AS. Knocking out RIPK3 provided a potential therapeutic target for Cd-caused macrophage polarization and subsequent AS.

**Graphical Abstract:**
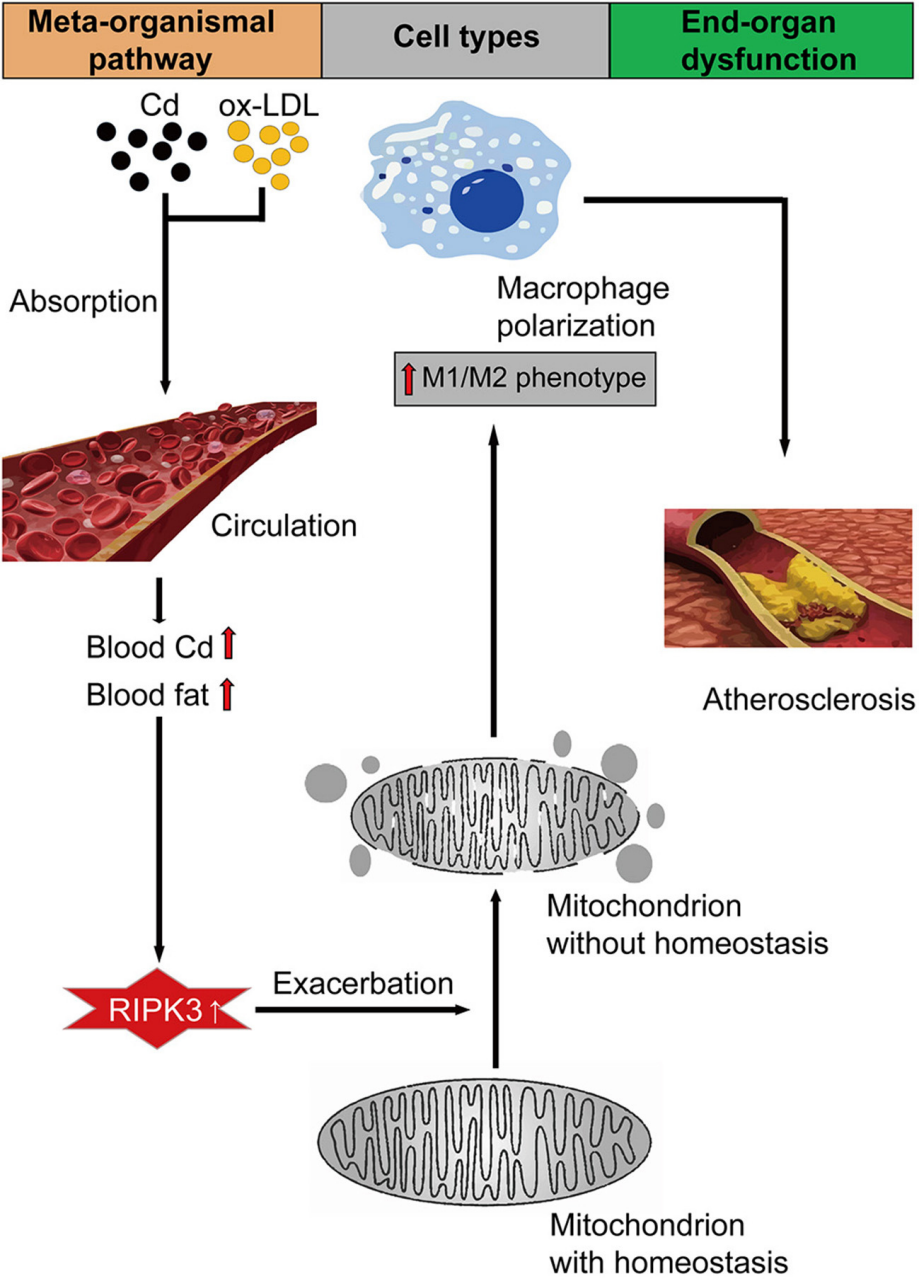
The mechanism of Cd-induced AS *via* RIPK3-regulated mitochondrial homeostasis.

## Introduction

With the rapid development of the industry, cadmium (Cd) emerged as a common heavy metal pollutant in the global environment. Because of the half-life of 10–35 years in humans and animals, Cd can cause serious damage to the body through biological amplification and accumulation (1). Evidence suggested that the vascular wall is a target of Cd deposition, whose concentration is up to 20 μM as compared to the blood concentration with a low nM range (2). A large body of cross-sectional studies has shown that Cd exposure is associated with cardiovascular diseases (CVDs), such as atherosclerosis (AS) (3), coronary heart disease (4), and peripheral arterial disease (PAD) (5). A recent study for the population found that blood and urinary Cd levels were positively correlated with the occurrence of AS plaques, and the content of Cd in the plaques was significantly higher than that in the blood (6). However, the exact mechanism of Cd-induced AS is not clear.

Macrophages are the largest number of immune cells in the cardiovascular system, with functional diversity and plasticity. The M1-type macrophage secretes pro-inflammatory factors, such as reactive oxygen species (ROS), tumor necrosis factor-alpha (TNF-α), and interleukin 1 beta (IL-1β), which are well documented to damage the AS plaque stability and cause stroke (7). On the contrary, the IL-10, FGF-1, IGF-1, TGF-β, and IL-12 released by the M2-type macrophage contribute to the enhancement of AS plaque stability (8). Additionally, the dynamics of the mitochondrial membrane are tightly coupled with the constant reshaping of the cellular mitochondrial network in a series of processes, involving organelle fusion and fission (division) as well as the ultrastructural remodeling of the membrane (9). Pieces of evidence accumulated have proved that the imbalance of the mitochondrial homeostasis is the initial factor of macrophage dysfunction, which emerges as the impairment of the protective autophagy (10) and promotes an inflammatory pathway (11). Given all these, it is meaningful to investigate whether Cd exposure affects the mitochondrial homeostasis of macrophages and further promotes the M1-type polarization of macrophages in AS, which has been seldomly studied until now.

In addition, as a member of the receptor-interacting protein (RIP) family of the serine/threonine protein kinases, the receptor-interacting serine/threonine kinase 3 (RIPK3) is closely related to inflammatory activation, which contributes to the expression of the nuclear Factor kappa-light-chain-enhancer of activated B (NF-KB) transcription factor (12). As we know, the inhibition of RIPK3 suppresses the RNA virus-induced activation of the NLRP3 inflammasome (13). On the aspect of membrane integrity, p-MLKL, phosphorylated by RIPK3 at the threonine 357 (Thr357) and serine 358 (Ser358) sites, transfers from the cytosol to the cell plasma membrane and the membranes of the organelles, where it directly disrupts membrane integrity (14). Additionally, RIPK1/RIPK3 complex directly phosphorylates Drp1 at the serine 616 site [p-Drp1^(Ser616)^] and triggers its translocation to the mitochondria, finally increasing the mitochondrial division (15). Prior studies have suggested that RIPK3-knock-out can reduce plaque formation in the advanced stage of AS (16). Therefore, whether RIPK3 is involved in the Cd-induced macrophage mitochondrial membrane dynamic imbalance and M1-type polarization in the progression of AS is the question to be studied in this research.

Thus, we hypothesized that a high-level Cd concentration in the blood could increase the expression of RIPK3-P-MLKL in the macrophages, and the RIPK3 inhibition would restrain the M1-type polarization of macrophages and subsequent AS mediated by Cd through restoring the macrophage mitochondrial homeostasis.

## Materials and Methods

### Data Availability

The data that support the findings of this study are available from the corresponding author upon reasonable request.

### Cell Culture

RAW264.7 macrophages were cultured in a Rosewell Park Memorial Institute (RPMI) 1640 medium supplemented with 20% fetal bovine serum (FBS), 100 U/ml penicillin, and 100 mg/ml streptomycin at 37°C in a humidified atmosphere with 5% carbon dioxide (CO_2_) and maintained in a logarithmic growth phase for all experiments. Then, the RAW264.7 macrophages were exposed to cadmium chloride (CdCl_2_) (GHTECH 1.09633.010) at different concentrations for 24 h. The first set was 0.0, 2.0, 4.0, 6.0, 8.0, 10.0, 20.0, 30.0, 40.0 and 50.0 μmol/L which was used for the cell counting kit-8 (CCK-8) to evaluate the cell viability of RAW264 macrophages exposed to CdCl_2_. The results from the CCK-8 showed that the cell viability of the RAW264.7 macrophages was more than 90% when exposed to CdCl_2_ with a concentration of ≤ 10 μmol/L. Furthermore, to know the optimal dose of CdCl_2_ within 10 μmol/L, the RAW264.7 macrophages were exposed to different concentrations of CdCl_2_ (0.0, 1.0, 5.0, and 10.0 μmol/L), and the results showed that 1 μmol/L CdCl_2_ had the most obvious pro-inflammatory effect. Therefore, CdCl_2_ (1 μmol/L) was used in the following experiment. The CdCl_2_ was ≥99.0% analytically pure and ≥98.0% chemically pure. To study the effect of mitochondrial homeostasis on the macrophages, we pretreated the RAW264.7 macrophages with 5 mM of N-Acetyl-L-cysteine (NAC) (Beyotime S0077) for 1 h to reduce the redox reactions or 50 μM of mitochondrial division inhibitor-1 (Mdivi-1) (ab144589) for 3 h to alleviate the mitochondria division. Additionally, bone marrow-derived macrophages (BMDMs) were isolated from the ApoE^−/−^/RIPK3^−/−^ mice to research the effects of RIPK3 on Cd-exposed macrophages.

### Animal Models of AS

The animal experiments were carried out according to the National Institutes of Health Guidelines on the Use of Laboratory Animals and were approved by the Animal Ethics Committee of the Southern Medical University. With the exposure to Cd-contaminated food or water, the blood levels of Cd at any point in time are typically in the range of 10–100 nmol/L (17). As a result, 7-week-old male ApoE^−/−^ mice, fed with high-fat diets or chow diets (provided by the Guangdong Medical Laboratory Animal Center) for 3 months, were exposed to Cd in different concentrations (100 mg/L). In that way, the concentration of the blood Cd in the mice was close to or a little more than 10–100 nmol/L. To study the effect of mitochondrial homeostasis on the formation of AS plaque, we administered NAC orally (20 mM) to the drinking water of the mice exposed to a high-fat with Cd diet. Besides, Mdivi-1 was dissolved in dimethylsulfoxide (DMSO) and was i.p. injected (50 mg/kg body weight) per day. The RIPK3-knockout mice with a C57BL/6 background were obtained from GemPharmatech, Nanjing, China, then bred with ApoE^−/−^mice to establish ApoE^−/−^/RIPK3^−/−^ mice. Mice from each group were killed at 19 weeks for tissue digestion (*n* = 5–7 per group).

### Tissue Collection and Analysis

After being fed with a high-fat or chow diet for to 3 months, the mice were killed with deep anesthesia for blood collection. Plasma was obtained from the blood samples of the mice by centrifugation (Thermo Scientific™ Medifuge™, Massachusetts, United States) at 1,000 × g for 15 min 4°C and then stored at −80°C. The mice were subsequently fixed by perfusion through the cardiac apex with a phosphate buffer saline (PBS), and the specimens of the aortic root were obtained without the peripheral adipose tissue.

### CCK-8 (Cytotoxicity Assay)

The RAW264.7 cells in the logarithmic growth phase were inoculated in a 96-well plate according to 10,000 cells/well. The next day, the macrophages were observed to grow up to 70%, then treated with different concentration gradients of CdCl_2_ for 24 h. The cell viability was detected by a CCK-8 kit purchased from Dojindo (CK04), Kumamoto, Japan, in a Plate Reader (Bio Tek Instruments Epoch™, Vermont, United States).

### RNA Extraction, Reverse Transcription, and Real-Time Quantitative Polymerase Chain Reaction (qRT-PCR)

The total RNA was extracted from the macrophages using a Trizol reagent (Invitrogen, Massachusetts, United States, 15596026). The RNA was quantified and reverse-transcribed into complementary DNAs using a PrimeScriptTM RT Master Mix kit (TaKaRa, Kusatsu, Shiga, Japan, RR036A). Finally, a quantitative real-time PCR analysis (qRT-PCR) was performed using a TB Green Premix Ex TaqTMII (TaKaRa RR820A) kit on a Light Cycler96 PCR instrument (Roche, Basel, Switzerland). The Vazyme cycling conditions were: 95°C for 30 s followed by 39 cycles at 95°C for 10 s and 60°C for 30 s. Then, a melting curve analysis was performed by increasing the temperature from 65 to 95°C for 15 min. Glyceraldehyde 3-phosphate dehydrogenase (GAPDH) was used as a loading control. The PCR primers used in this study were synthesized using TSINGKE (Beijing, China) and the sequences were: GAPDH [CATCACTGCCACCCAGAAGACTG (F), ATGCCAGTGAGCTTCCCGTTCAG (R)], IL-1β [TGGACCTTCCAGGATGAGGACA (F), GTTCATCTCGGAGCCTGTAGTG (R)], IL-6 [TACCACTTCACAAGTCGGAGGC (F), CTGCAAGTGCATCATCGTTGTTC (R)], IL-10 [CGGGAAGACAATAACTGCACCC (F), CGGTTAGCAGTATGTTGTCCAGC (R)], TGF-β [CGAAGCGGACTACTATGCTAAA (F), TCCCGAATGTCTGACGTATTG (R)], CD86 [ACGTATTGGAAGGAGATTACAGCT (F), TCTGTCAGCGTTACTATCCCGC (R)], CD206 [GTTCACCTGGAGTGATGGTTCTC (F), AGGACATGCCAGGGTCACCTTT (R)].

### Western Blot

The proteins were extracted from the cells, tissues, or mitochondria, while the mitochondria were isolated from the RAW264.7 macrophages or aortic root using a cell mitochondria isolation kit (Beyotime C3601, Beyotime Biotechnology, Beijing, China) or tissue mitochondria isolation kit (Beyotime C3606). After the proteins were measured using BCA (Beyotime Biotechnology) to the same concentration in each group, we boiled the proteins for 10 min, and separated them using 10% sodium dodecyl sulfate-(SDS)-polyacrylamide gels then transferred onto a polyvinylidene difluoride (PVDF) membrane. After blocking the non-specific binding sites with 5% non-fat milk in Tris-buffered saline-Tween 20 (TBS-T), we incubated the membranes overnight with antibodies. The following primary antibodies were used: rabbit polyclonal antibody against GAPDH (ab9485), rabbit polyclonal antibody against NLRP3 (ab214185), rabbit monoclonal antibody against IL-1 beta[EPR16805-15] (ab234437), rabbit monoclonal antibody against TNF alpha [EPR19147] (ab183218), rabbit monoclonal antibody against Opa1[EPR11057(B)] (ab157457), rabbit monoclonal antibody against MLKL (phospho S345) [EPR9515(2)] (ab196436), and rabbit monoclonal antibody against RIP3 (phospho S232) [EPR9516(N)-25] (ab195117), all from Abcam (Cambridge, United Kingdom). The rabbit monoclonal antibody against Phospho-NF-kappaB p65 (Ser536) (93H1) (Cell Signaling, #3033S) and rabbit monoclonal antibody against COX IV (D6I4K) (Rodent Specific) (Cell Signaling, #38563) were from Cell Signaling Technology (Dancers, Massachusetts, United States). The rabbit polyclonal antibody against LC3I/II (WL01506) and rabbit polyclonal antibody against Drp1 (WL03028) were from Wanlei Biology, Shenyang, China. After having been incubated with the corresponding secondary antibodies (Boster BA1054) for 1.5 h at room temperature, the immunoblot bands were detected using enhanced chemiluminescence (Engreen 29100, Engreen Inc., San Jose, California, United States). Prestained molecular weight marker proteins (Thermo#26616) were used to calculate the molecular weights of proteins. The membranes were exposed to autoradiography film following the incubation with an enhanced chemiluminescence imaging system (Engreen 29100). The signal intensities were checked using the Gel-Pro Analyzer 4.0 software (Media Cybernetics, Silver Spring, Maryland, United States).

### Enzyme-Linked Immunosorbent Assay (ELISA)

The levels of the inflammation markers in the cell supernatant or mouse plasma, including interleukin 1β (IL-1β) and interleukin 6 (IL-6), were examined using ELISA kits (DAKEWE #1210122 #1210602, Shenzhen, China).

### Flow Cytometry

The cell surfaces were stained with a phycoerythrin (PE)-conjugated anti-mouse F4/80 antibody (Biolegend 123110, California, United States), APC-conjugated anti-mouse CD206 (MMR) antibody (Biolegend 141708, California, United States), and FITC-conjugated anti-mouse CD86 antibody (Biolegend 105006, California, United States). The expression of F4/80 was determined using flow cytometry to identify the macrophages. The expression of CD86 was used to delineate the M1 macrophages, while the cells stained by the APC-conjugated anti-mouse CD206 (MMR) antibody could be identified as M2 macrophages. The cell suspensions were stained for 30 min on ice with specific antibodies and washed two times with 3 ml of PBS buffer supplemented with 0.5% bovine serum albumin (BSA). Finally, we analyzed it using flow cytometry (CytoFlex A00-1-1102, California, United States).

### Immunofluorescence

The cultured Raw264.7 macrophages or aortic root tissues were fixed with 4% paraformaldehyde, incubated in 1% Triton for 10 min and then in 2% BSA for 1 h, successively incubated with primary antibodies overnight at 4°C, followed by incubation with secondary antibodies. The following primary antibodies were used: F4/80 (BM8.1) rat mAb (#71299S) from Cell Signaling Technology, CD86 Polyclonal rabbit antibody (13395-1-AP), CD206 Polyclonal rabbit antibody (18704-1-AP), Tom20 Polyclonal rabbit antibody (11802-1-AP), and LC3B-Specific Polyclonal rabbit antibody (18725-1-AP) from Proteintech, Chicago, United States. Then, the samples were stained with 6-diamidino-2-phenylindole (DAPI) to visualize the nucleus and observed by a laser confocal microscope (LEICA SP8). All the images and staining intensities were acquired and measured using analysis software (Image J).

### Mitochondrial Function Detection

The mitochondrial superoxide of the Raw264.7 macrophages was detected using a MitoSOX™ Red mitochondrial superoxide indicator (Invitrogen™ M36008), and the mitochondrial membrane potential was determined *via* a mitochondrial membrane potential assay kit with JC-1 (Beyotime C2006) according to the instructions of the manufacturer.

### Transmission Electron Microscope (TEM) Observations

The macrophages growing to the logarithmic phase were scraped and collected to be fixed with glutaraldehyde for 4 h. Then, sections (60 nm) were cut and stained with lead citrate and uranyl acetate at room temperature for 4 h. Next, samples were embedded in resin at room temperature for another 2 h. A Hitachi H-7500 Transmission Electron Microscope (Hitachi, Ltd., Tokyo Japan) was used to observe the samples.

### Oil Red O Staining

After the serial 10 μm sections were cut, the optimal cutting temperature (OCT) compound-embedded tissues were stained with Oil Red O (Solarbio 08010) to evaluate the lipid content, and Re-dyed with hematoxylin (Beyotime), differentiated by hydrochloric alcohol (Beyotime), washed by double-distilled water, and finally observed under a microscope (OLYMPUS, Shinjuku City, Tokyo, Japan).

### Quantification of Blood Cd

We added 500 μl 1% HNO3 to 250 μl of the whole blood of mice and added 0.01% TritonX-100 (Amresco 0694) to 5 ml. Then we measured the concentration of the blood Cd using an ICP-Mass Spectrometer (PerkinElmer NexIONTM 350X).

### Statistical Analyses

Data were generated through at least three independent experiments and were presented as the means ± SEM, then analyzed with the method of *t*-test between two groups or one-way ANOVA followed by a Bonferroni comparison test among three groups. Statistical analyses were carried out using Prism 8 (GraphPad, San Diego, California, United States). A two-tailed *P* < 0.05 was considered to be statistically significant.

## Results

### Cd Promoted Macrophage Polarization to a Pro-inflammatory Phenotype and Atherosclerosis

**Figure 1:**
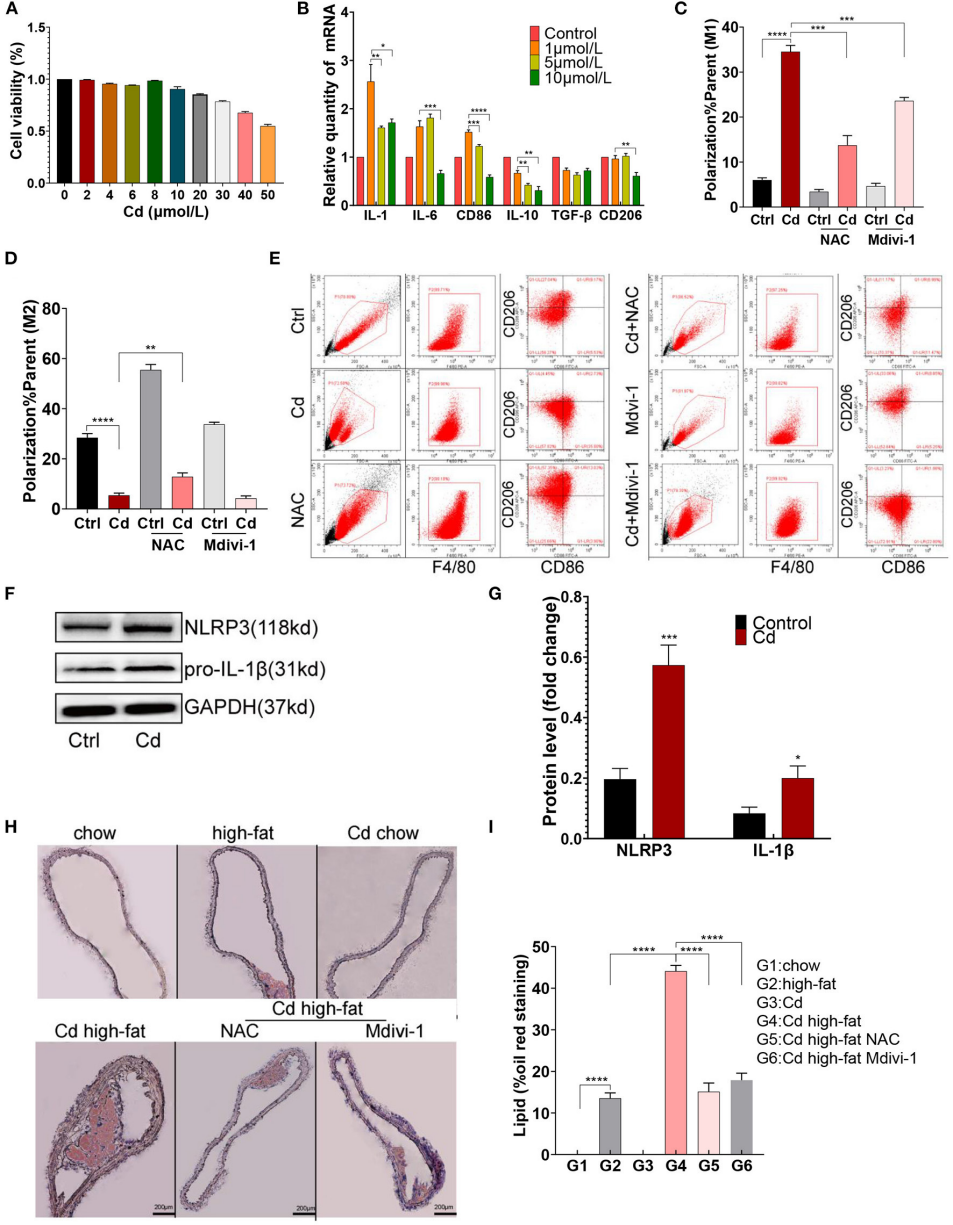
M1-type polarization of macrophage and AS caused by cadmium (Cd). **(A)** The survival rate of the macrophages exposed to different concentrations of Cd was detected by the CCK-8 kit. **(B)** The expression of the messenger RNA (mRNA) related to Cd-induced inflammation was measured by quantitative PCR (q-PCR). **(C–E)** The Cd-induced polarization of macrophages showed by flow cytometry. **(F,G)** The protein expression levels of the NLR family pyrin domain containing 3 (NLRP3) and pro-IL-1β in the RAW264.7 cells after treatment with Cd. **(H,I)** Representative photomicrographs of the aortic arch segments from the Cd- and high-fat-treated ApoE^−/−^ mice stained with Oil Red O (*n* = 5–7 per group). Data are shown as mean ± SD. ^*^p < 0.05, ^**^p < 0.01, ^***^p < 0.001, ^****^p < 0.0001.

To evaluate the cytotoxicity caused by Cd, CCK8 measurement was performed after exposing the RAW264.7 macrophages to CdCl_2_. The cell viability of the RAW264.7 macrophages was more than 90% when exposed to CdCl_2_ with a concentration of ≤ 10 μmol/L (Figure 1A). Subsequently, the macrophages were treated with 0.0, 1.0, 5.0, and 10.0 μmol/L CdCl_2_ for the qRT-PCR detection of related inflammatory factors, and the results showed that a lower concentration of CdCl_2_ (1 μmol/L) had the most obvious pro-inflammatory effect (Figure 1B). To further verify the effect of Cd on macrophage polarization in AS, 1 μmol/L Cd exposure was used for subsequent assays, including flow cytometry, western blot (WB), and ELISA after the RAW264.7 was foamed with ox-LDL (50 μg/ml) *in vitro*. Upon being exposed to Cd, the surface-specific molecule of the M1-type macrophages, CD86, was markedly upregulated while the surface-specific molecule of the M2-type macrophages, CD206, was significantly downregulated (Figures 1C–E). Then, the results of the WB showed that the expressions of NLRP3 and pro-IL-1β (Figures 1F,G), and pNF-KB and TNF-α (Supplementary Figures 1A,B), were increased after Cd exposure. Moreover, the inflammatory factor, IL-6, in the supernatant of macrophages was increased after the Cd exposure, measured using an ELISA kit (Supplementary Figure 1C). The proteins mentioned above were closely connected with the M1-type polarization of macrophages (18). Finally, the Oil red O staining of the aortic root revealed that the plaque area of the high-fat feeding group with Cd exposure was significantly increased compared with those without Cd exposure (Figures 1H,I). Taken together, Cd promoted macrophage polarization toward the pro-inflammatory phenotype and subsequent AS.

### The Level of RIPK3 Was Increased in Cd-Mediated Atherosclerosis

**Figure 2:**
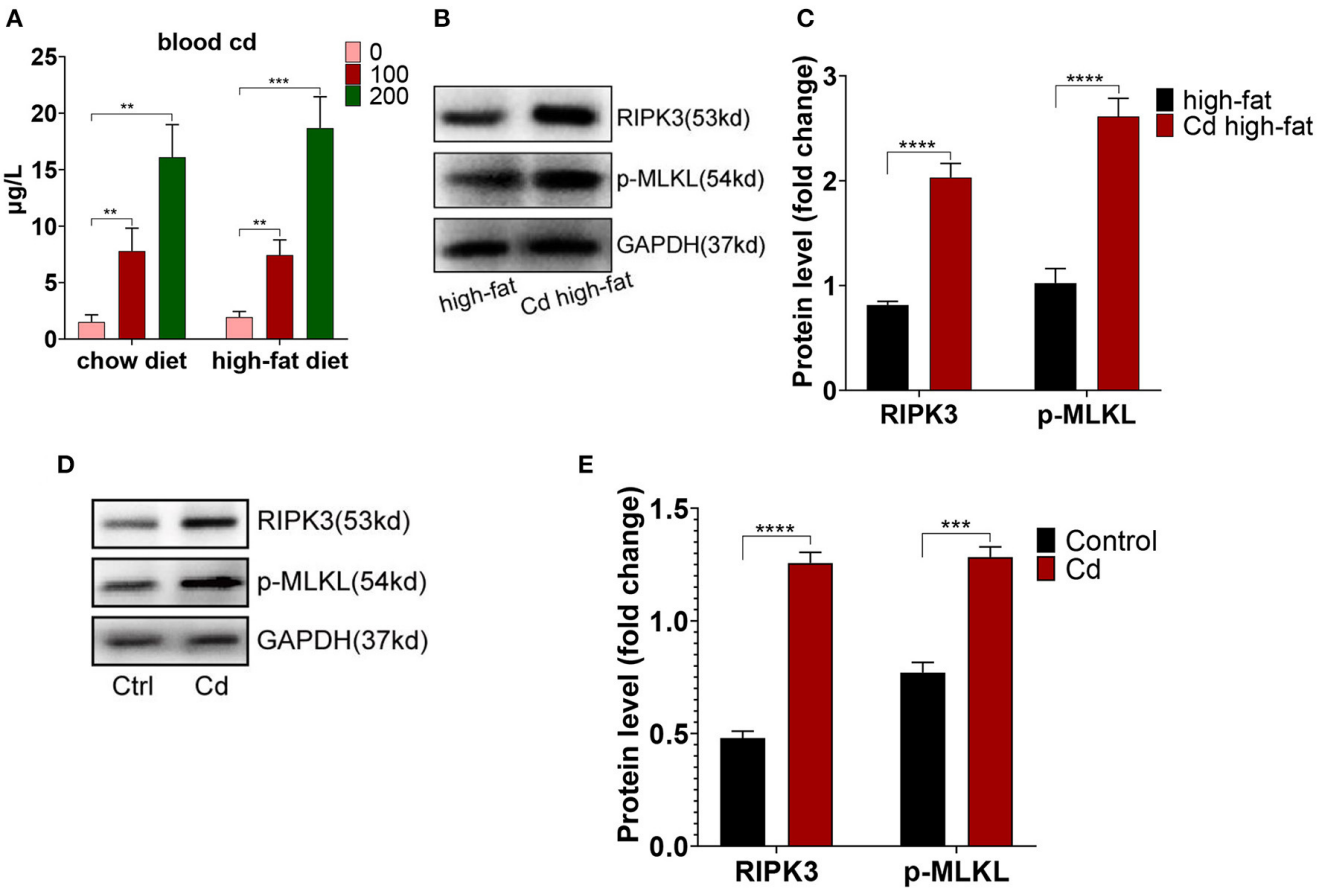
The increased level of the receptor-interacting protein kinase 3 (RIPK3) in Cd-mediated atherosclerosis. **(A)** The concentration of blood Cd after Cd exposure. The protein expression levels of RIPK3 and pseudokinase-mixed lineage kinase domain-like protein (p-MLKL) *in vivo* **(B,C)** and *in vitro* **(D,E)** (*n* = 5–7 per group). Data are shown as mean ± SD. ^**^p < 0.01, ^***^p < 0.001, ^****^p < 0.0001.

To further research the mechanism of Cd-mediated AS, ApoE^−/−^ mice fed up with a high-fat diet were exposed to Cd *in vivo*. The results showed that the blood Cd concentration was proportional to the Cd exposure level. The blood Cd of the group exposed to the 100 mg/L concentration of Cd is closer to the blood Cd of a human body exposed to Cd pollution (10–100 nmol/L) (17) (Figure 2A). Meanwhile, we found that the expression of RIPK3 and its downstream protein, p-MLKL, were significantly increased under Cd exposure both *in vivo* (Figures 2B,C) and *in vitro* (Figures 2D,E).

### Deletion of RIPK3 Inhibited Polarity Shift Toward Inflammatory Macrophages and Atherosclerosis

**Figure 3:**
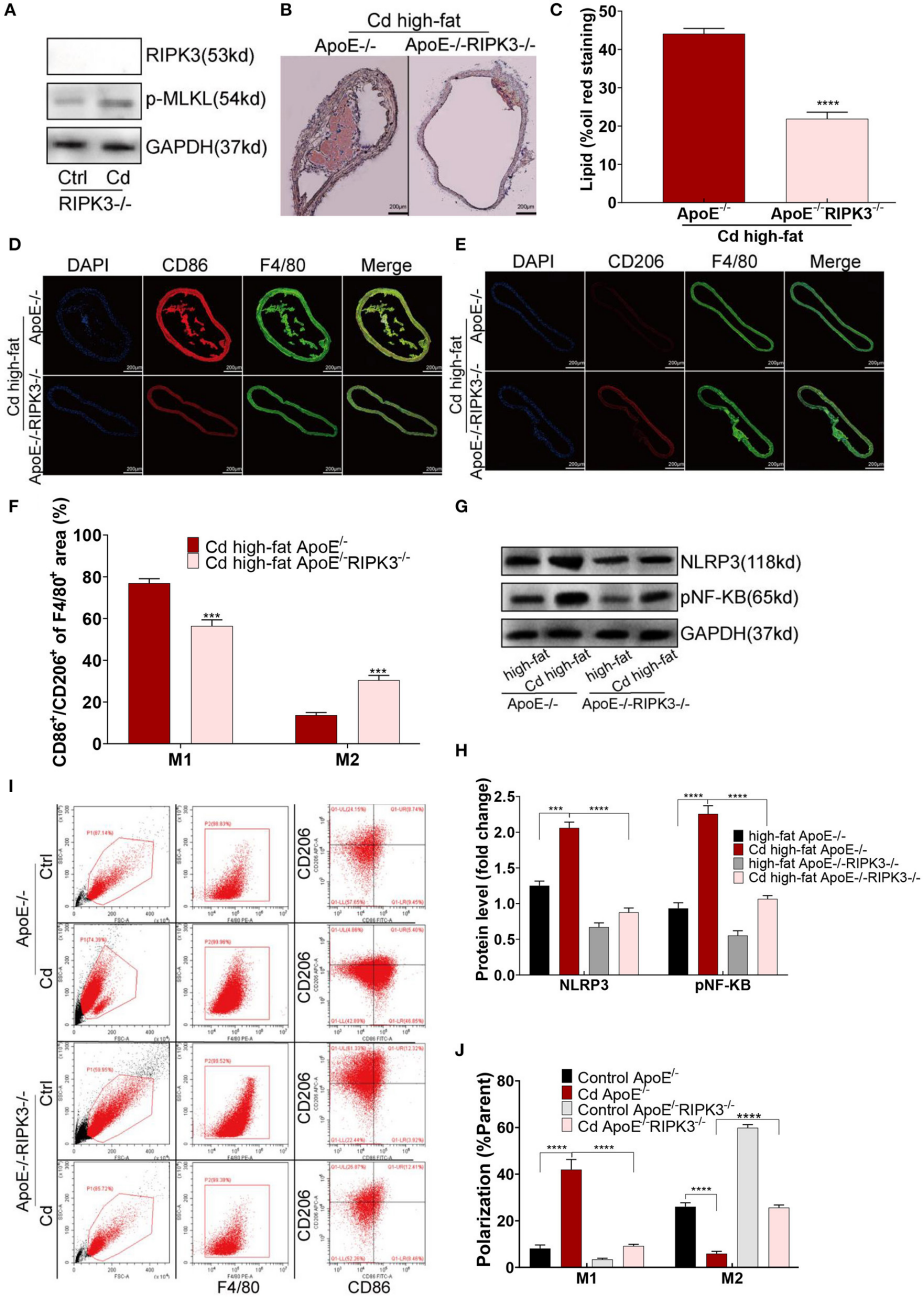
Deletion of the RIPK3 inhibited polarity shift toward inflammatory macrophages and atherosclerosis. **(A)** The protein expression levels of RIPK3 and p-MLKL in the ApoE^−/−^/RIPK3^−/−^mice. **(B)** Representative photomicrographs of the aortic arch segments from the Cd- and high-fat-treated ApoE^−/−^ or ApoE^−/−^/ RIPK3^−/−^ mice stained with Oil Red O. **(C)** Quantification of the atherosclerotic lipid content. **(D–F)** Aortic arch of the Cd- and high-fat-treated ApoE^−/−^ or ApoE^−/−^/ RIPK3^−/−^ mice were probed with specific antibodies against the macrophage marker F4/80 and co-probed with antibodies against the markers of M1 (CD86) or the markers of M2 (CD206). **(G,H)** The protein expression levels of NLRP3 and pNF-KB in the aortic root of the ApoE^−/−^ or ApoE^−/−^/ RIPK3^−/−^ mice after treatment with Cd. **(I,J)** The polarization of BMDMs from ApoE^−/−^ or ApoE^−/−^/ RIPK3^−/−^ mice treated by Cd shown by flow cytometry (*n* = 5–7 per group). Data are shown as mean ± SD. ^***^p < 0.001, ^****^p < 0.0001.

To explore the effect of RIPK3 on Cd-induced AS, we knocked out this protein to breed ApoE^−/−^RIPK3^−/−^ mice (Figure 3A). The mice were fed with a high-fat diet and exposed to Cd. The Oil red O staining of the aortic root showed that the area of the AS plaques were significantly decreased in the ApoE^−/−^/RIPK3^−/−^ mice compared with the ApoE^−/−^ mice (Figures 3B,C). The immunofluorescence staining of the aortic roots showed that the level of M1-type polarization (CD86^+^/F4/80^+^) decreased while the level of M2-type polarization (CD206^+^/F4/80^+^) increased in the ApoE^−/−^/RIPK3^−/−^ mice compared with the ApoE^−/−^ mice (Figures 3D–F). The expressions of NLRP3 and pNF-KB were downregulated in the aortic root (Figures 3G,H). Additionally, the deletion of RIPK3 inhibited the polarity shift toward the M1-type BMDMs extracted from the ApoE^−/−^/RIPK3^−/−^, as shown by flow cytometry (Figures 3I,J). The expressions of NLRP3 and pNF-KB were inhibited in the BMDMs of the ApoE^−/−^/RIPK3^−/−^ mice (Supplementary Figures 2A,B). The plasma level of the inflammatory cytokines, IL-1β and IL-6, secreted by the M1-type macrophages were significantly decreased in the ApoE^−/−^/RIPK3^−/−^ mice (Supplementary Figure 1C). These results indicated that the deletion of RIPK3 attenuated Cd-induced inflammatory responses and subsequent AS.

### RIPK3 Knockout Enhanced Mitochondrial Homeostasis and Protective Autophagy Disrupted by Cd

**Figure 4:**
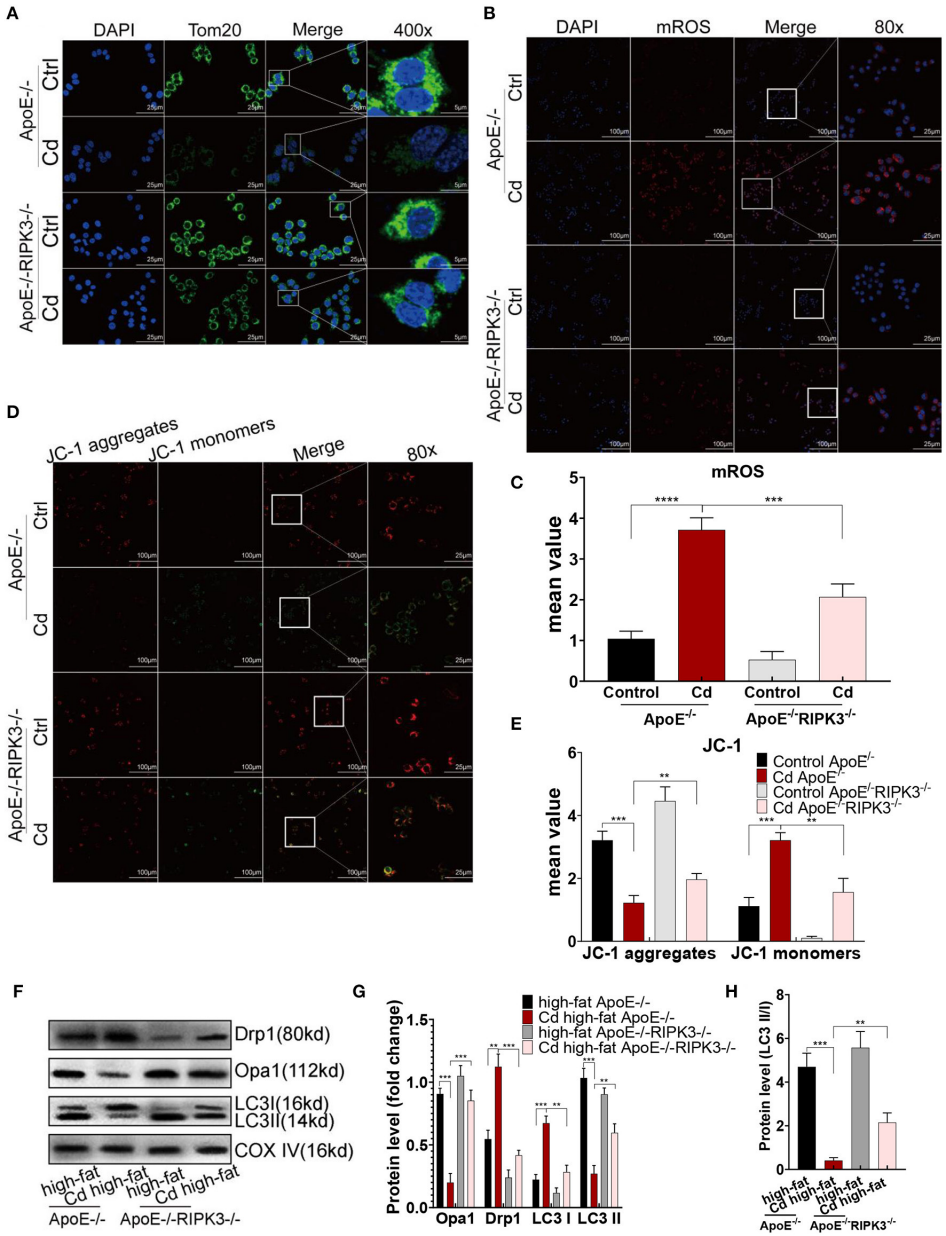
The RIPK3-p-MLKL pathway regulated the mitochondrial homeostasis in the Cd-induced AS. **(A)** Immunofluorescence images of mitochondrial membrane protein, Tom20, in the bone marrow-derived macrophages (BMDMs) of the ApoE^−/−^ or ApoE^−/−^/RIPK3^−/−^ mice treated with Cd. **(B,C)** Immunofluorescence images and fluorescence intensity of the mitochondrial superoxide (mROS) in the BMDMs. **(D,E)** Mitochondrial membrane potential stained with a fluorescent probe (JC-1). **(F,G)** The protein expression levels of Opa1, Drp1, LC3I, and LC3II in the mitochondria of the aortic arch in the ApoE^−/−^ or ApoE^−/−^/RIPK3^−/−^ mice treated with Cd. COX-IV is the internal reference of the mitochondrial protein. **(H)** The ratio of LC3II and LC3I indicating the autophagy level (*n* = 5–7 per group). Data are shown as mean ± SD. ^**^p < 0.01, ^***^p < 0.001, ^****^p < 0.0001.

To investigate the potential mechanism of RIPK3, we performed immunofluorescence staining on the macrophages with the mitochondrial membrane protein Tom20 antibody. The results showed that mitochondrial fragmentation was greatly reduced in the ApoE^−/−^/RIPK3^−/−^ mice compared with the ApoE^−^/^−^ mice under Cd exposure (Figure 4A). In addition, the results from the MitoSOX™ Red mitochondrial superoxide indicator showed that the level of mitochondrial superoxides (mROS) was significantly decreased in the BMDMs from the ApoE^−/−^/RIPK3^−/−^ mice compared with those from the ApoE^−^/^−^ mice (Figures 4B,C). Moreover, the RIPK3 knockout enhanced the mitochondrial membrane potential in Figures 4D,E. The WB results revealed that Cd exposure led to the increased expression of the mitochondrial division protein Drp1, decreased fusion protein Opa1, and decreased protective autophagy (the ratio of LC3II/I) both in BMDMs (Supplementary Figures 3C–E) and *in vivo* (Figures 4F–H). RIPK3 knockout restored the effects above. Furthermore, we performed an immunofluorescence staining with autophagy-related protein LC3II, and the results showed that the protective autophagosomes in BMDMs were significantly decreased under Cd exposure while RIPK3 knockout enhanced the protective autophagy (Supplementary Figures 3A,B). These results indicated that RIPK3 knockout significantly reversed the Cd-disrupted mitochondrial homeostasis.

### Improving Mitochondrial Homeostasis Through RIPK3 Inhibition Restored the Balance of Macrophage Polarization *in vitro*

**Figure 5:**
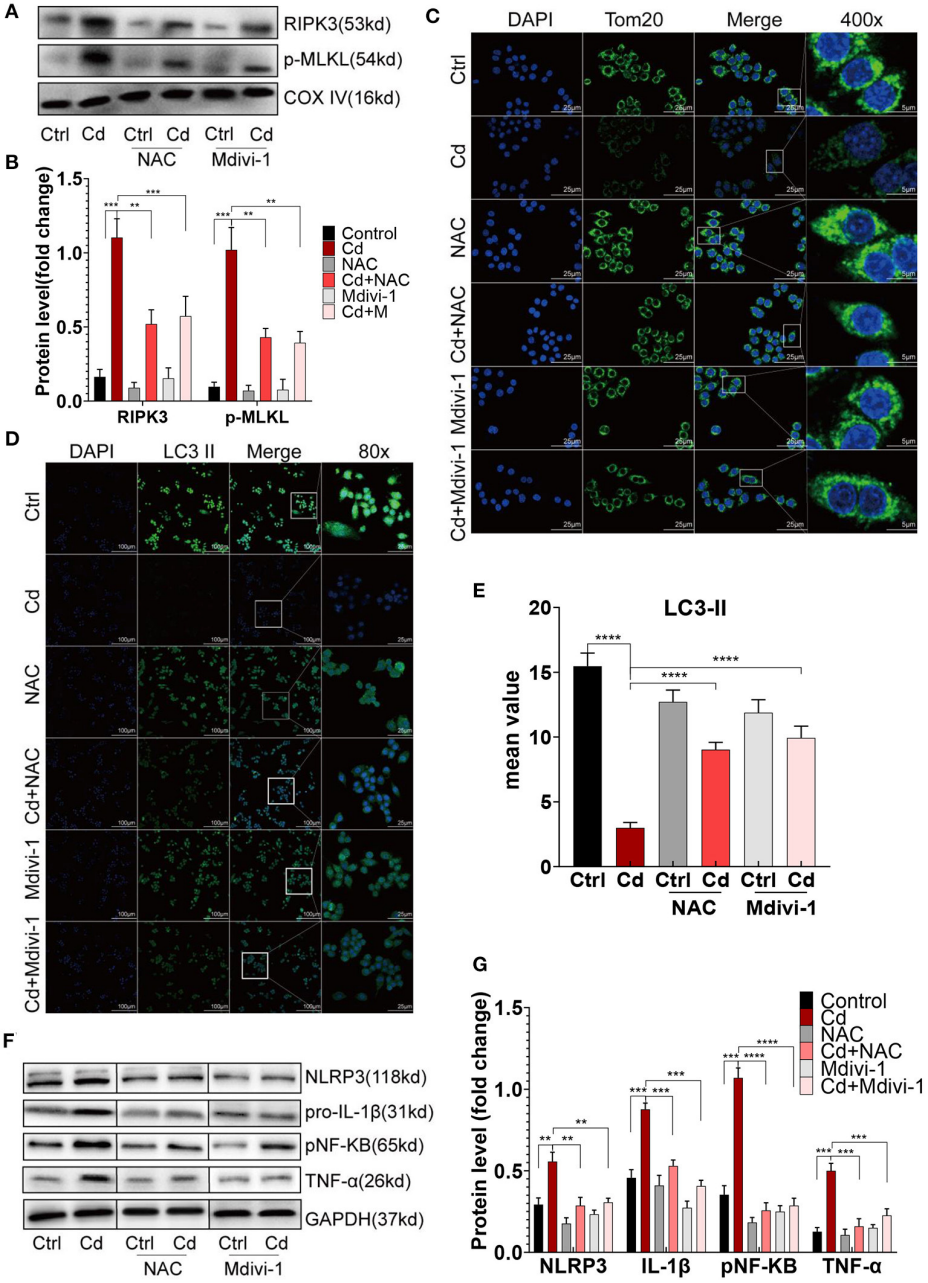
Improving mitochondrial homeostasis through RIPK3 inhibition restored the balance of the macrophage polarization *in vitro*. **(A,B)** The protein expression levels of RIPK3 and p-MLKL in RAW264.7 treated with Cd, NAC, and Mdivi-1. **(C)** Immunofluorescence images of the mitochondrial membrane protein, Tom20, in the RAW264.7 cells. **(D,E)** Immunofluorescence images and fluorescence intensity of LC3II in the RAW264.7 cells. **(F,G)** The protein expression levels of NLRP3, pro-IL-1β, pNF-KB, and TNF-α in the RAW264.7 treated with Cd, N-Acetyl-L-cysteine (NAC), and mitochondrial division inhibitor-1 (Mdivi-1). Data are shown as mean ± SD. ^**^p < 0.01, ^***^p < 0.001, ^****^p < 0.0001.

To improve mitochondrial homeostasis, the antioxidant NAC was used to alleviate the mROS production, and Mdivi-1, a mitochondrial fission inhibitor, was used to inhibit the expression of Drp1. First, the expression of RIPK3 in the mitochondria was downregulated after the NAC or Mdivi-1 treatment (Figures 5A,B). The results showed that NAC and Mdivi-1 effectively reduced the mitochondrial fragments of the Cd-exposed macrophages (Figure 5C), decreased the production of mROS (Supplementary Figures 4A,B), and recovered the mitochondrial membrane potential (Supplementary Figures 4C,D), indicating the restoration of mitochondrial homeostasis. Additionally, results from the WB identified that NAC or Mdivi-1 decreased the expression of Drp1, and increased the expression of Opa1 and the ratio of LC3II/I (Supplementary Figures 4E–G). Meanwhile, increased autophagosomes were presented using immunofluorescence with an LC3II antibody after the NAC and Mdivi-1 treatment (Figures 5D,E). Similarly, the results from the TEM showed that the treatment with NAC and Mdivi-1 significantly increased the content of autophagosomes in macrophages, while the production of mitophagy was more obvious in the Mdivi-1-intervened group (Supplementary Figure 5A). The decreased expression of CD86 and the increased expression of CD206 were both demonstrated using flow cytometry with the recovery of the mitochondrial homeostasis (Figure 1E). Moreover, NAC and Mdivi-1 markedly decreased the expression of NLRP3, pNF-KB, and their downstream proteins, pro-IL-1β and TNF-α (Figures 5F,G). These results indicated that improving mitochondrial homeostasis with NAC or Mdivi-1 effectively reversed Cd-induced macrophage polarization toward the pro-inflammatory phenotype via RIPK3 inhibition.

### Maintaining Mitochondrial Homeostasis by NAC or Mdivi-1 Counteracted Atherosclerosis *in vivo*

**Figure 6:**
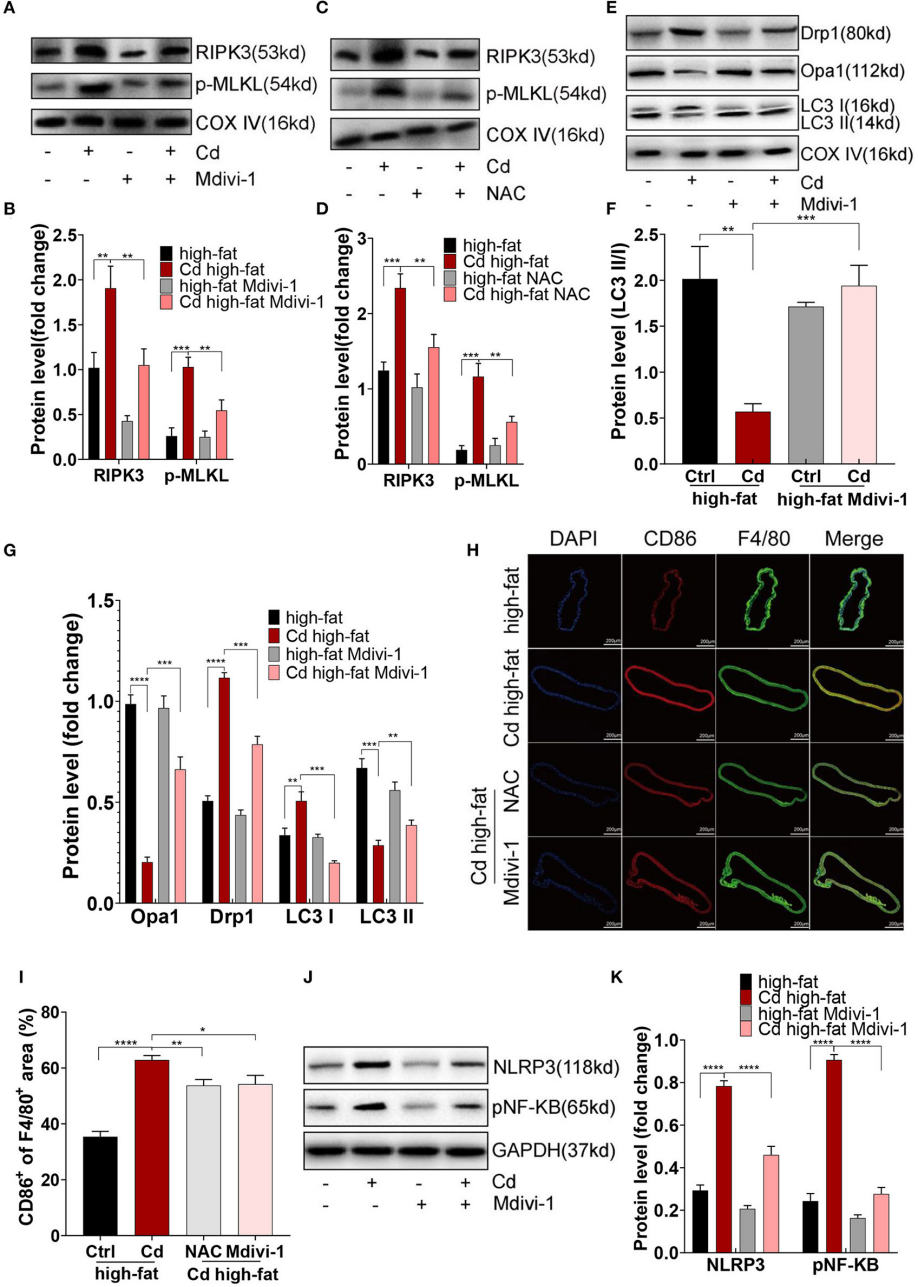
Mitochondrial homeostasis mediated inflammation of Cd-caused AS *in vivo*. **(A–D)** The protein expression levels of RIPK3 and p-MLKL in the ApoE^−/−^ mice treated with Cd, NAC, and Mdivi-1. **(E–G)** The protein expression levels of Opa1, Drp1, LC3I, and LC3II in the mitochondria of the aortic arch in the ApoE^−/−^ mice after the treatment with Cd, high-fat, and Mdivi-1. **(H)** Aortic arch of the Cd-, high-fat-, NAC-, and Mdivi-1-treated ApoE^−/−^ mice were probed with specific antibodies against the macrophage marker F4/80 and co-probed with antibodies against the markers of M1 (CD86). **(I)** Quantification of CD86^+^ of the F4/80^+^ area in the aortic arch. **(J,K)** The protein expression levels of NLRP3 and pNF-KB in the mice aortic arch (*n* = 5–7 per group). Data are shown as mean ± SD. ^*^p < 0.05, ^**^p < 0.01, ^***^p < 0.001, ^****^p < 0.0001.

First, the expression of RIPK3 and its downstream protein, p-MLKL, were significantly downregulated after the treatment with NAC or Mdivi-1 (Figures 6A–D). The Oil red O staining of the aortic root revealed that NAC and Mdivi-1 effectively reduced the plaque area in the mice with high-fat and Cd exposure (Figures 1H,I). Additionally, the mice treated with NAC or Mdivi-1 showed a significant increase in the expression of Opa1 and the ratio of LC3II/I but a remarked decrease in the expression of Drp1 (Figures 6E–G, Supplementary Figures 6A–C). The treatment with NAC or Mdivi-1 significantly decreased the M1-type polarization (the ratio of CD86^+^/F4/80^+^; Figures 6H,I), while increasing the M2-type polarization (Supplementary Figures 6D,E). Moreover, the expression of NLRP3 and pNF-KB decreased after the treatment with NAC or Mdivi-1 (Figures 6J,K, Supplementary Figures 6F,G). The results from ELISA further confirmed that the levels of the M1-type inflammatory markers, il-1β and il-6, were significantly decreased after the treatment with NAC or Mdivi-1 (Supplementary Figures 6H,I). Taken together, maintaining the mitochondrial homeostasis with NAC or Mdivi-1 could reverse Cd-induced macrophage polarization to the pro-inflammatory phenotype and thus counteract AS *in vivo*.

## Discussion

Our findings showed that Cd disrupted the macrophage mitochondrial homeostasis through the RIPK3 signaling pathway and caused the M1-type polarization of the macrophage, aggravating the progression of AS (Graphical Abstract). The detection of mitochondrial homeostasis proved that Cd exposure induced more mitochondrial fragmentation, and mROS while the mitochondrial membrane potential and protective autophagy were reduced by Cd. Knocking out RIPK3 could reverse the effects above. To the best of our knowledge, we were the first to demonstrate that the mechanism of Cd-induced AS is the imbalance of the macrophage mitochondrial homeostasis *via* the RIPK3 pathway.

Previous studies on Cd-induced inflammation of macrophages reported that oral exposure to Cd (3 h) triggered an acute inflammatory response in the intestines of the mice, initiated by the over-expression of the tissue macrophage inflammatory protein-2 messenger RNA (mRNA) which was produced by both the intestinal epithelial cells and macrophages. But this study showed no significant changes in the cytokines, IL-1β, and TNF-α (19). In contrast, Ninkov et al. (20) studied the effects of oral Cd exposure on intestinal immunity, suggesting that Cd consumption resulted in the changes of the intestinal flora due to the reduction of Lactobacillus strain, and intestinal inflammation due to the increasing pro-inflammation cytokines (TNF, IL-1β, IFN-γ, IL-17). Furthermore, Kielldahl et al. (21) found that blood Cd was associated with pro-inflammatory macrophage density in the sections of carotid plaques with the most frequent rupture which was previously shown to contain most Cd. The studies mentioned above indicated that Cd can induce an inflammatory response in macrophages which was the same as the result of this article. However, the pro-inflammatory properties of Cd are still controversial. Till now, the previous studies that mentioned the effect of Cd on macrophage polarization are very limited. One research in 2016 revealed that in patients with chronic obstructive pulmonary disease (COPD), Cd exposure inhibited the NF-kB pathway, dose-dependently inhibited LPS-induced immunoreaction by macrophages and inhibited M1-type macrophage behavior with less effect on M2-type polarization. This article became new evidence that Cd could lead to immune dysfunction, further increasing the susceptibility to COPD infection (22). More recently, Yao et al. (23) proved in 2021 that Cd activated the oxidative stress-mediated TLR4/NF-KB/NLRP3 inflammatory signal transduction, leading to porcine adrenal fibrosis by promoting macrophage polarization toward M1. However, there is still little research about the mechanism of Cd-induced AS through macrophage polarization. Furthermore, another study came up to be the first report investigating the effects of Cd on the inflammatory responses and oxidative stress together *in vitro* system, and the results showed that murine macrophage exposed to Cd significantly decreased the inflammatory responses but increased the oxidative stress, a little different from studies explained above (24). The difference may be because Cd concentration and treatment time that high-level Cd exposure for a long time can impair cell viability and function so that the high concentration of Cd in the lungs of smokers impairs the normal pro-inflammatory function of macrophages and worsens the infection of COPD. However, the concentration of the blood Cd is not high, as a result, the function of the macrophages in the cardiovascular system has not been impaired.

As with the mechanism of Cd-induced inflammation, an existing study on Cd-induced liver injury showed that liver injury was mediated by inflammatory mediators activated by ROS (25). Glycine, an antioxidant, could reduce the secretion of inflammatory cytokines IL-6, TNF-α, and IL-1 by the macrophage U937 cells (26). In addition, another study has shown that the enhanced REDOX reaction in peritoneal macrophages of the mice exposed to chronic low levels of Cd-induced lipid peroxidation, and increased the expressions of cyclooxygenase-2 and inducible nitric oxide synthase, finally, causing inflammatory response (27). Therefore, we can conclude that Cd-induced oxidative stress is related to inflammatory response, and the former is the upstream pathway of the latter which is the same as the result of our research. Furthermore, the main sources of intracellular ROS are membrane-derived and mitochondrion-derived. The former is mainly dependent on nicotinamide adenine dinucleotide (NADPH) oxidase, while the latter is associated with mitochondrial membrane potential damage. As to the mitochondrion-derived ROS, studies have shown that the mitochondria in THP-1 macrophages could be targets of Cd toxicity: Cd bound to thiol proteins on the mitochondrial membrane, and then changed the mitochondrial membrane permeability to inhibit the mitochondrial respiratory chain response, or damaged the function of electron transfer chain complex III, and finally induced the production of ROS (28). However, the mechanism of mitochondrial damage and subsequent macrophage polarization involved in the process of Cd-induced AS remain unclear.

Additionally, it has been reported that RIPK3 plays an important role in necroptosis, which is regarded as a highly pro-inflammatory form of cell death (29). A sustained mitochondrial homeostasis imbalance would trigger the necroptosis of macrophages (11). As for the effect of RIPK3 on CVD, Zhang et al. reported in 2018 that in hyperlipidemic patients, the plasma level of RIPK1, RIPK3, and MLKL were obviously increased (30). It was demonstrated in another research that RIPK3 was primarily activated by ischemia-reperfusion injury and then upregulated PGAM5 expression, CypD phosphorylation, which obligated endothelial cells to undergo necroptosis *via* augmenting the mPTP (mitochondrial permeability transition pore) opening (31). However, there has been no study revealing that Cd could impair macrophage mitochondrial homeostasis and promote macrophage polarization contributing to atherosclerosis *via* regulating RIPK3 signaling. In this study, we filled this gap in knowledge by reporting that chronic Cd exposure impaired macrophage mitochondrial homeostasis and promoted macrophage polarization contributing to AS through RIPK3 pathway.

The molecular mechanism of inflammatory reaction includes the discovery by Hyun et al. (32) that 20–60 μM concentration of CdCl_2_ significantly induced the increase of the IL-8 secretion through the activation of the NF-KB in human intestinal epithelial cell Caco-2. Moreover, *in vitro* studies showed that Cd activated the NF-KB pathway through increasing oxidative stress levels (33). In addition, the team of Whir has successively found that the activation of heat shock response induced by Cd effectively protected the lung function of the mice. Compared with HSF^−/−^ mice, the NF-KB pathway was suppressed in wild-type mice, leading to lower infiltrating macrophages and neutrophils (34, 35). These studies showed that the activation of NF-KB was one of the molecular mechanisms by which Cd promoted inflammation, the same as our results. Besides, the team of Kunpeng Wu found that the M1-type polarization of macrophages required the activation of the NLRP3 inflammasome (18). But no researches related to Cd-induced macrophage polarization have paid attention to NLRP3 inflammasome except this study.

In addition to AS, Cd exposure can also worsen other CVD progression. It has been reported that Cd-induced hypertension results in decreased endothelial nitric oxide synthase protein level (36). Additionally, macrophages create a beneficial microenvironment for the survival of myocardial cells, which is crucial for the survival and regeneration of myocardial cells after MI (37). Local inflammatory response and changes in the immune microenvironment are considered to be one of the main reasons for the difficulty in myocardial repair and regeneration after myocardial tissue injury (38). Therefore, the influence of Cd exposure on the immune microenvironment in the heart region may be the key interference factor in the myocardial repair after MI in the Cd-exposed area. At the same time, many known studies have reported that the bone and kidney were two of the important target organs of multi-organ damage caused by Cd poisoning, among which osteoporosis is the main manifestation of bone damage caused by Cd (39). Moreover, existing studies have shown that with the growth of age, the lost human calcium from bones would be deposited to the cardiovascular system and then induce vascular calcification (40). Although there is a strong relationship between osteoporosis and vascular calcification, there is a lack of research on the mechanism of vascular calcification caused by Cd exposure.

Finally, in addition to the environmental pollutant, Cd, which was the focus of this paper, other heavy metal pollution, such as lead, mercury, and arsenic, may also have an impact on the cardiovascular system (4). Will they have a synergistic effect with Cd? Is there a natural substance (e.g., selenium) that can counteract the damage caused by Cd contamination? Further study needs to be investigated.

## Conclusion

Our findings demonstrated that mitochondrial homeostasis played an important role in the Cd-induced inflammatory response in the macrophages. We further revealed that RIPK3 regulated the Cd-induced mitochondrial homeostasis imbalance *via* Drp1 protein, leading to an increase in the expressions of NF-KB and NLRP3 proteins. Finally, Cd exposure induced M1-type polarization of macrophages in the cardiovascular system which was conducive to AS. These results revealed a novel mechanism for Cd-induced inflammation and offered new insights into the pathophysiology of AS caused by heavy metal pollution.

## Data Availability Statement

The original contributions presented in the study are included in the article/Supplementary Material, further inquiries can be directed to the corresponding author.

## Ethics Statement

The animal study was reviewed and approved by Animal Ethics Committee of Southern Medical University.

## Author Contributions

JZ: conceptualization, methodology, validation, investigation, data curation, writing—original draft, and visualization. WF: methodology, validation, writing—review and editing, and data curation. ML: methodology and validation. PC and XN: methodology. CO: methodology, validation, supervision, writing—review and editing, and funding acquisition. MC: methodology, conceptualization, supervision, writing—review and editing, and funding acquisition. All authors contributed to the article and approved the submitted version.

## Funding

This study was supported by the National Natural Science Foundation of China (Nos. 31771099, 81871504, 31771060, and 81971765).

## Conflict of Interest

The authors declare that the research was conducted in the absence of any commercial or financial relationships that could be construed as a potential conflict of interest.

## Publisher's Note

All claims expressed in this article are solely those of the authors and do not necessarily represent those of their affiliated organizations, or those of the publisher, the editors and the reviewers. Any product that may be evaluated in this article, or claim that may be made by its manufacturer, is not guaranteed or endorsed by the publisher.

## Abbreviations

AS: Atherosclerosis
Cd: Cadmium
CVD: Cardiovascular disease
Drp1: Dynamin-related protein 1
ELISA: Enzyme-linked immunosorbent assay
IL-1β: Interlukin 1β
Mdivi-1: Mitochondrial division inhibitor 1
NAC: N-Acetyl-L-cysteine
NF-KB: Nuclear Factor Kappa B
NLRP3: NLR Family Pyrin Domain Containing 3
Opa1: OPA1 Mitochondrial Dynamin Like GTPase
RIPK3: Receptor interacting serine/threonine kinase 3
TGF-α: transforming growth factor α
WB: Western blot.
