# Supplementary material for: Receptor-Interacting Protein Kinase 3 Inhibition Prevents Cadmium-Mediated Macrophage Polarization and Subsequent Atherosclerosis via Maintaining Mitochondrial Homeostasis
Source: Front Cardiovasc Med. 2021 Nov 8;8:737652. doi: 10.3389/fcvm.2021.737652 (PMC8606644; doi:10.3389/fcvm.2021.737652)
Supplement: Supplementary file 1 [file Data_Sheet_1.PDF]

# Supplemental data

**RIPK3 inhibition prevents cadmium-mediated macrophage polarization and subsequent atherosclerosis via maintaining mitochondrial homeostasis**

Jiexin Zhang[a,b]†, Weijing Feng[a,b,c]†, Minghui Li[a,b], Peier Chen[a,b], Xiaodong Ning[a,b] , Caiwen Ou*[a,b], Minsheng Chen*[a,b]

†These authors have contributed equally to this work and share first authorship.

[a]Department of Cardiology, Laboratory of Heart Center, Zhujiang Hospital, Southern Medical University, Guangzhou 510280, China

[b]Guangdong Provincial Key Laboratory of Shock and Microcirculation, Guangzhou 510280, China

[c]Department of Cardiology, State Key Laboratory of Organ Failure Research, Nanfang Hospital, Southern Medical University, Guangzhou 510515, China

DOI [to be added]

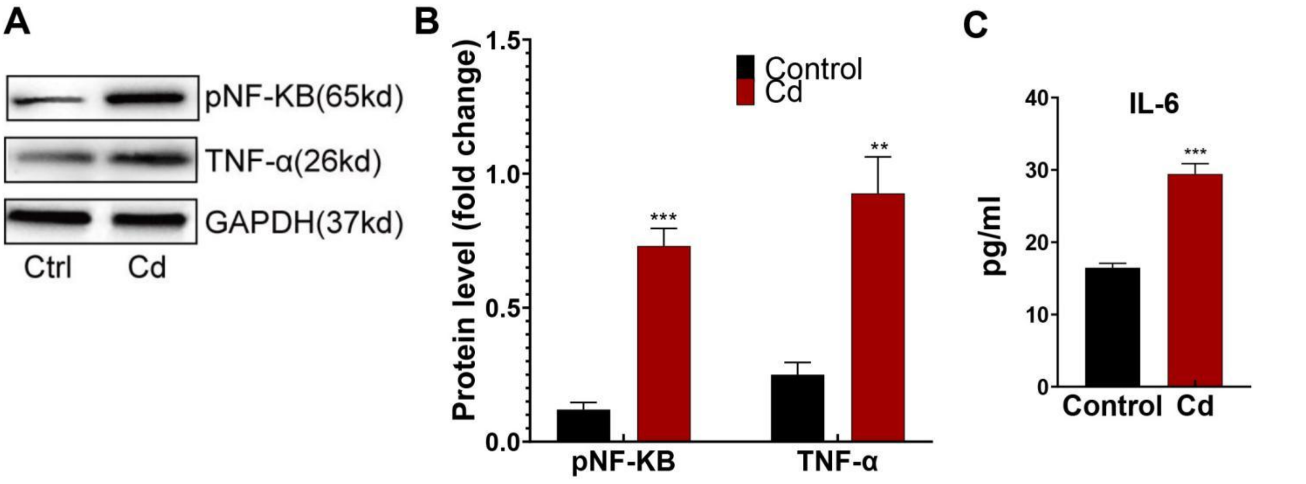


**Supplemental Figure S1**.M1-type polarization of macrophage caused by Cd. (A, B) The protein expression levels of pNF-KB and TNF-α in RAW264.7 cells after treatment with Cd. (C) The level of IL-6 in the supernatant of macrophages.

DOI [to be added]

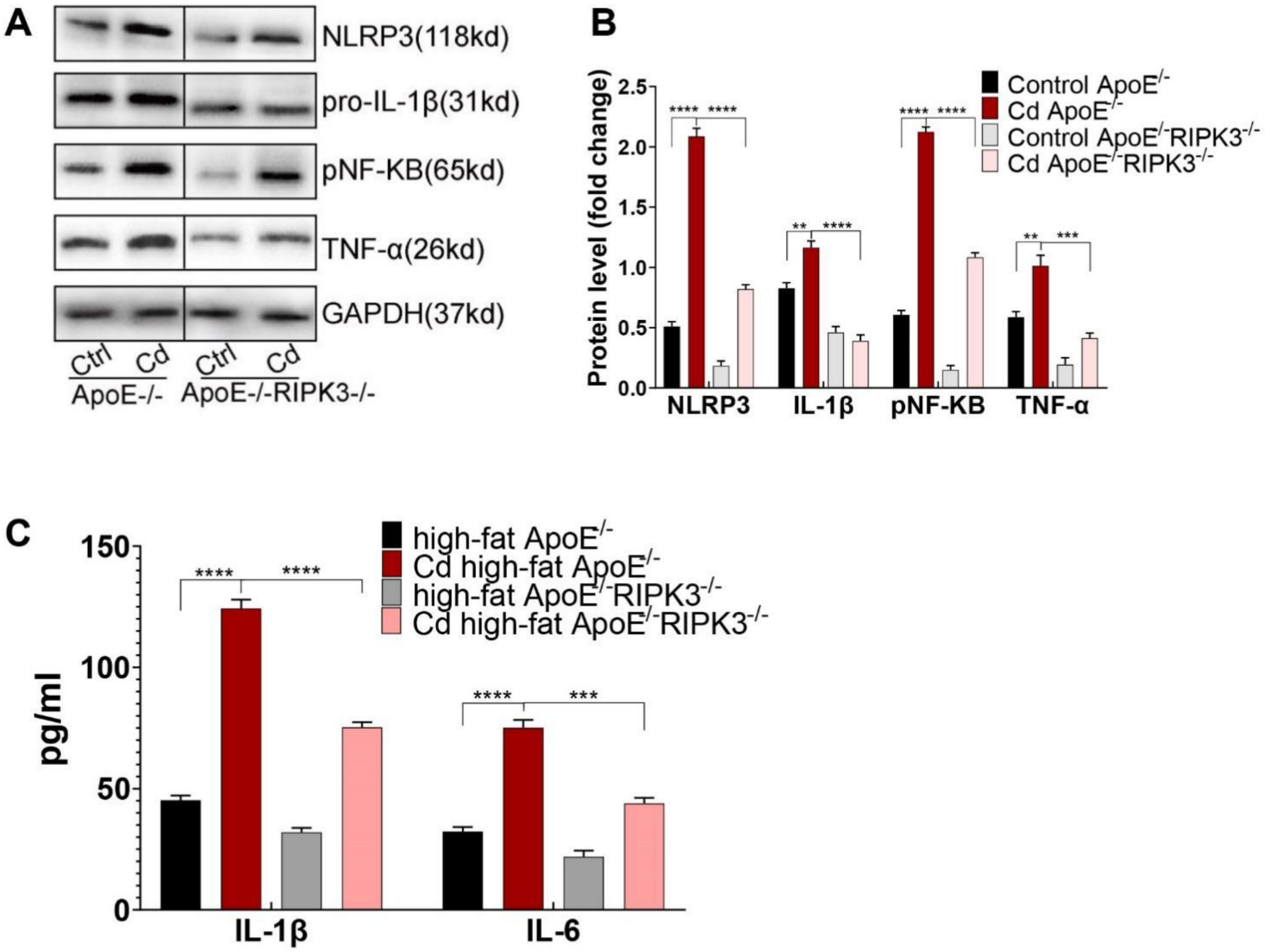


**Supplemental Figure S2**. Deletion of RIPK3 inhibited polarity shift toward inflammatory macrophages. (A, B) The protein expression levels of NLRP3, pro-IL-1β and pNF-KB, TNF-α in BMDMs from ApoE$^{-/-}$ or ApoE$^{-/-}$/ RIPK3$^{-/-}$ mice treated by Cd. (C) The concentration of IL-1β and IL-6 in plasma from ApoE$^{-/-}$ or ApoE$^{-/-}$/ RIPK3$^{-/-}$ mice treated by Cd. (n = 5 - 7 per group). Data are shown as mean ± SD. *p < 0.05, **p < 0.01, ***p < 0.001, ****p < 0.0001.

DOI [to be added]

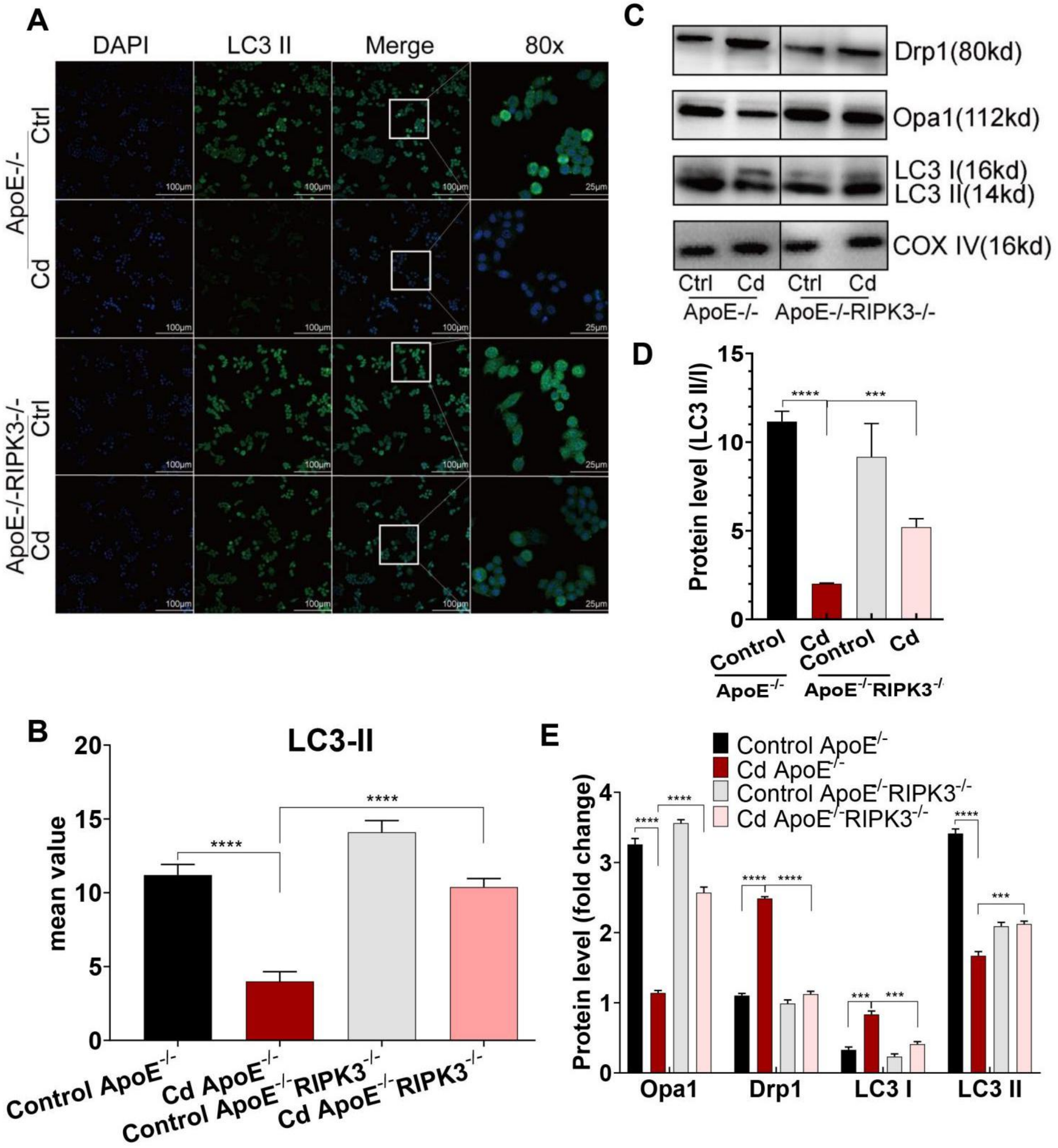


**Supplemental Figure S3.** RIPK3 knockout enhanced mitochondrial homeostasis and protective autophagy disrupted by Cd. (A, B) Immunofluorescence images and fluorescence intensity of LC3II in BMDMs from ApoE$^{-/-}$ or ApoE$^{-/-}$/ RIPK3$^{-/-}$ mice treated by Cd. (C-E) The protein expression levels of Opa1, Drp1, LC3I and LC3II in mitochondria of BMDMs treated with Cd. The ratio of LC3II and LC3I indicating the autophagy level. (n = 5 - 7 per group). Data are shown as mean ± SD. *p < 0.05, **p < 0.01, ***p < 0.001, ****p < 0.0001.

DOI [to be added]

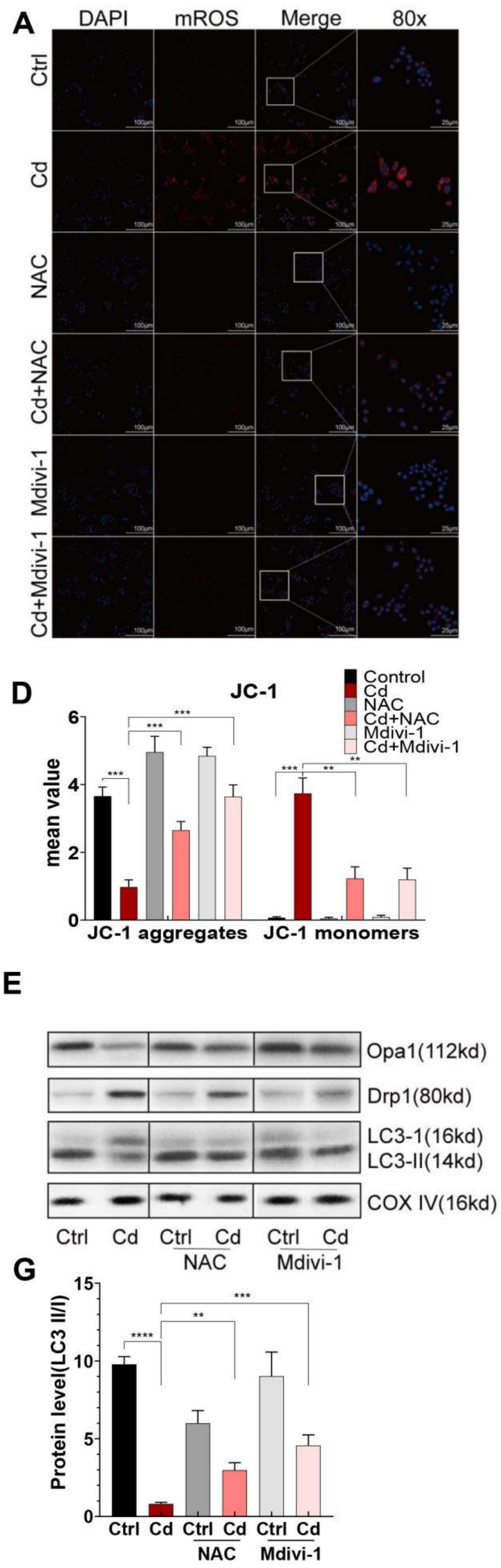


DOI [to be added]

**Supplemental Figure S4**. Improving mitochondrial homeostasis through RIPK3 inhibition. (A, B) Immunofluorescence images and fluorescence intensity of mitochondrial superoxide (mROS) in RAW264.7. (C, D) Mitochondrial membrane potential stained with fluorescent probe (JC-1) in RAW264.7. (E, F) The protein expression levels of Opa1, Drp1, LC3I and LC3II in mitochondria of RAW264.7 cells. (G) The ratio of LC3II and LC3I indicating the autophagy level. Data are shown as mean ± SD. *p < 0.05, **p < 0.01, ***p < 0.001, ****p < 0.0001.

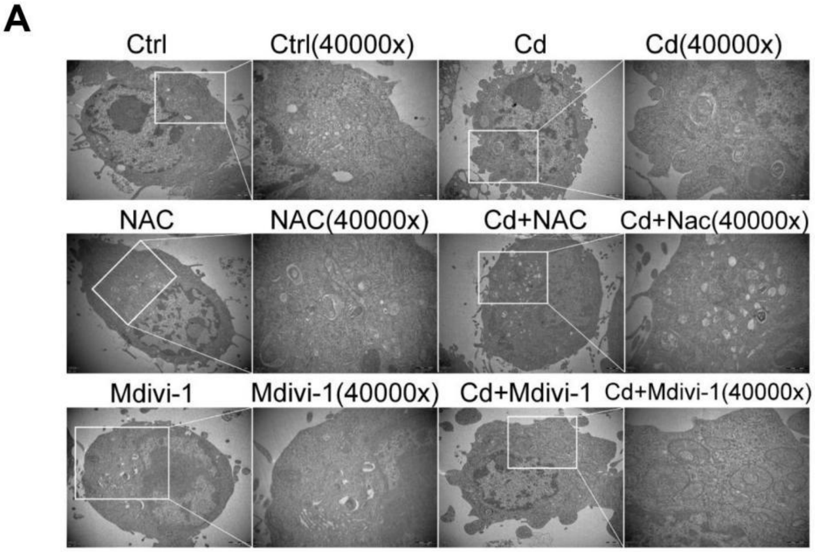


**Supplemental Figure S5.** Improving mitochondrial homeostasis through RIPK3 inhibition. (A) Representative TEM images of autophagosome in RAW264.7 cells after treatment with Cd, NAC and Mdivi-1. Data are shown as mean ± SD. *p < 0.05, **p < 0.01, ***p < 0.001, ****p < 0.0001.

DOI [to be added]

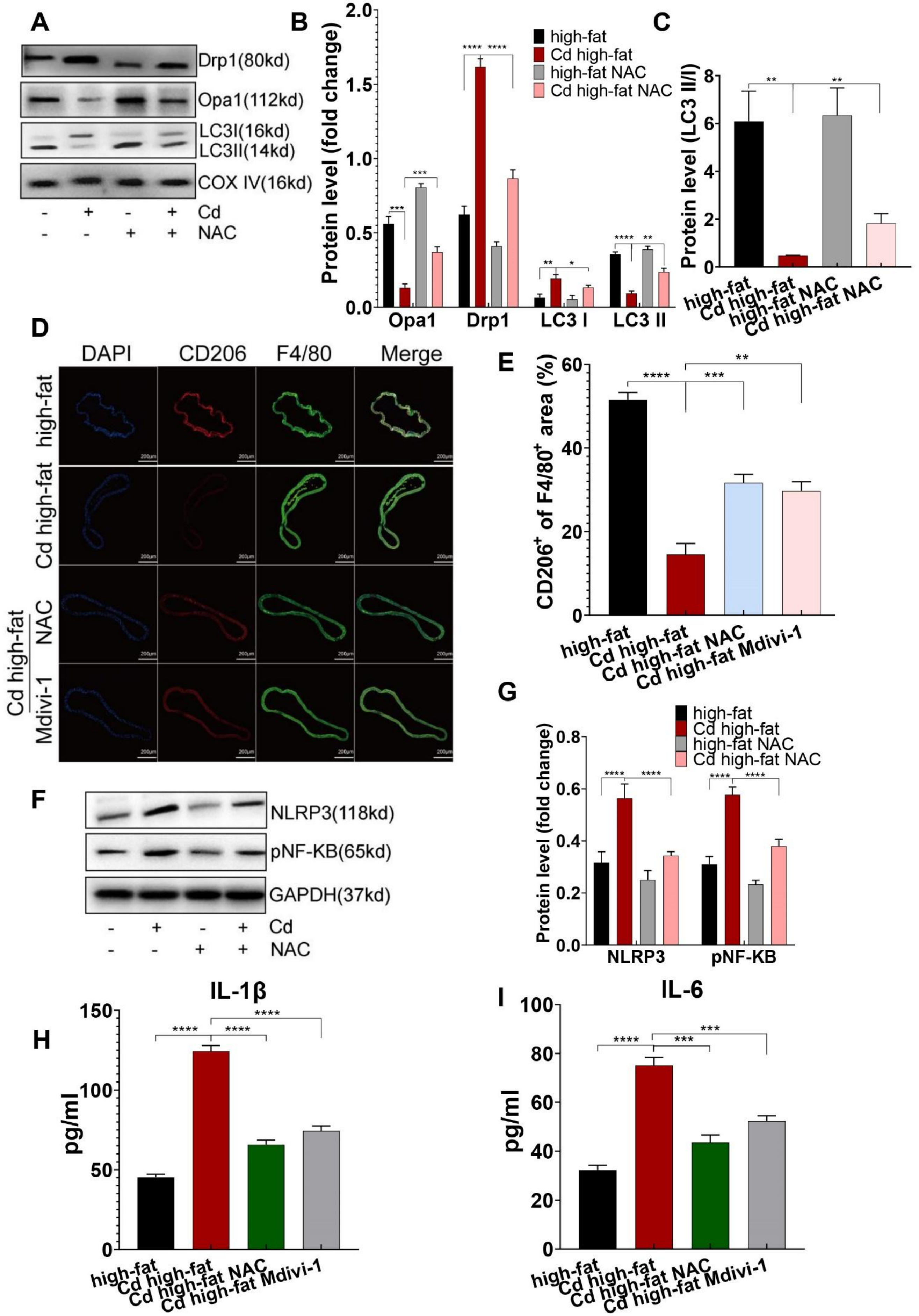

A
Drp1(80kd)
Opa1(112kd)
LC3I(16kd)
LC3II(14kd)
COX IV(16kd)
Cd
NAC
B
Protein level (fold change)
high-fat
Cd high-fat
high-fat NAC
Cd high-fat NAC
Opa1
Drp1
LC3 I
LC3 II
C
Protein level (LC3 II/I)
high-fat
Cd high-fat
high-fat NAC
Cd high-fat NAC
D
DAPI
CD206
F4/80
Merge
high-fat
Cd high-fat
Cd high-fat NAC
Cd high-fat Mdivi-1
E
CD206+ of F4/80+ area (%)
high-fat
Cd high-fat
Cd high-fat NAC
Cd high-fat Mdivi-1
F
NLRP3(118kd)
pNF-KB(65kd)
GAPDH(37kd)
Cd
NAC
G
Protein level (fold change)
high-fat
Cd high-fat
high-fat NAC
Cd high-fat NAC
NLRP3
pNF-KB
H
IL-1β
pg/ml
high-fat
Cd high-fat
Cd high-fat NAC
Cd high-fat Mdivi-1
I
IL-6
pg/ml
high-fat
Cd high-fat
Cd high-fat NAC
Cd high-fat Mdivi-1

**Supplemental Figure S6.** Maintaining mitochondrial homeostasis by NAC or Mdivi-1 counteracted atherosclerosis in vivo. (A-C) The protein expression levels of Opa1, Drp1, LC3I and LC3II in mitochondria of aortic arch in ApoE-/- mice after treatment with Cd, high-fat, and NAC. (D) Aortic arch of Cd - , high-fat - , NAC- and Mdivi-1-treated ApoE-/- mice were probed with specific antibodies against the macrophage marker F4/80 and co - probed with antibodies against the markers of M2 (CD206). (E) Quantification of CD206+ of F4/80+ area in aortic arch. (F, G) The protein expression levels of NLRP3 and pNF-KB in mice aortic arch. (H, I) The concentration of IL-1β and IL-6 in plasma from ApoE-/- mice treated by Cd, NAC and Mdivi-1. ($n = 5 - 7$ per group). Data are shown as mean ± SD. $^{*}p < 0.05$, $^{**}p < 0.01$, $^{***}p < 0.001$, $^{****}p < 0.0001$.

DOI [to be added]

Figure 1F

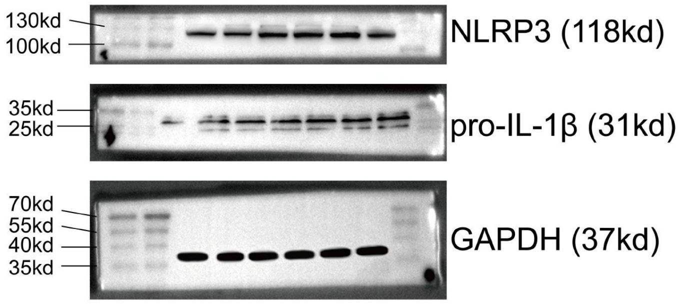


Figure 2B

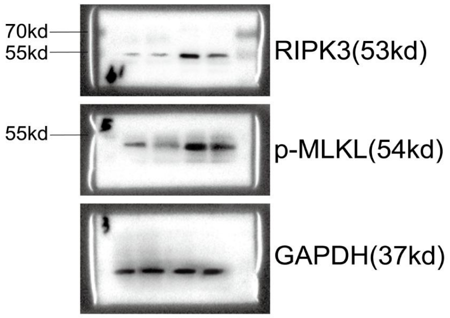


Figure 2D and Figure 3A

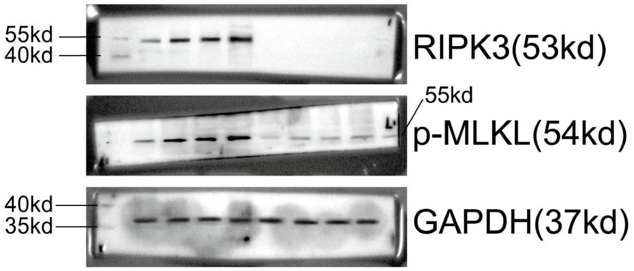


Figure 3G

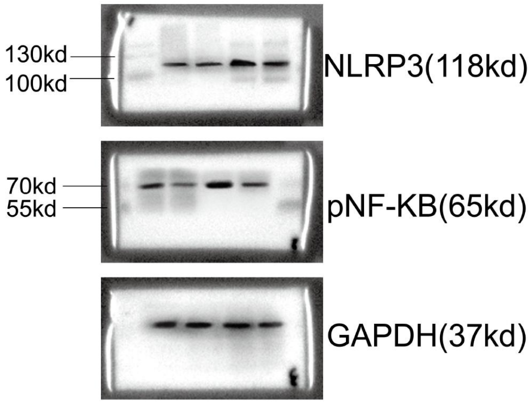


Figure 4F

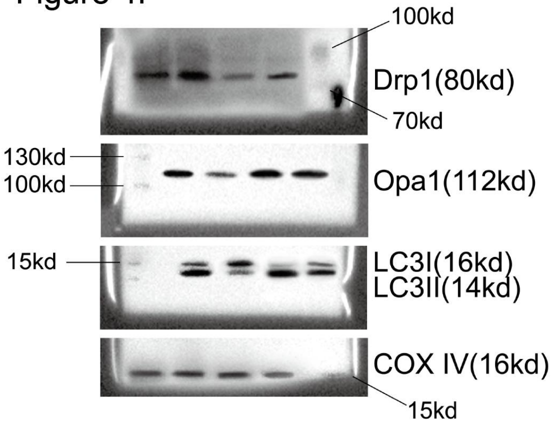


Figure 5A

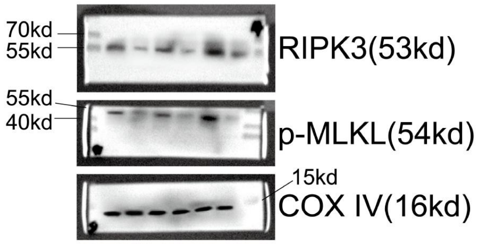


Figure 5F

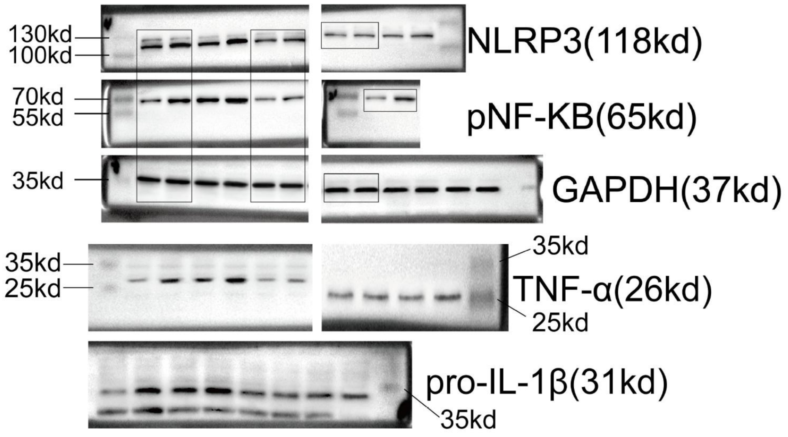


DOI [to be added]

Figure 6A

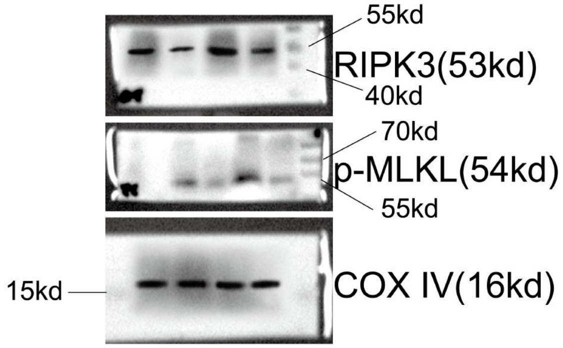


Figure 6C

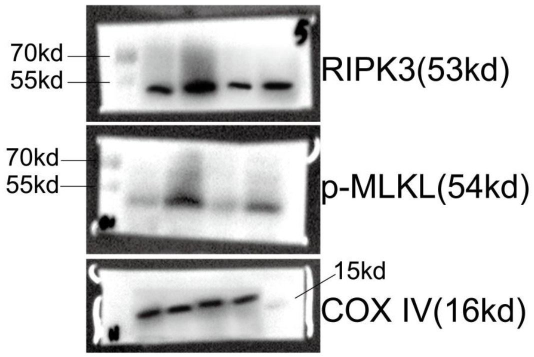


Figure 6E

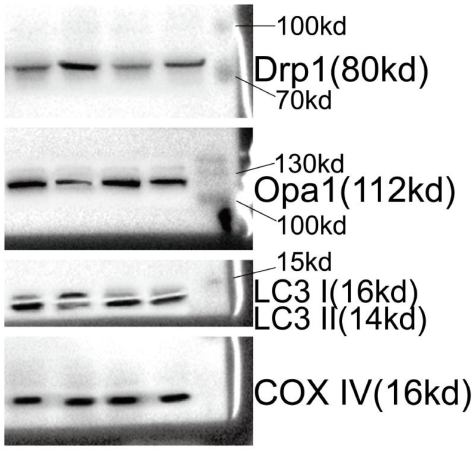


Figure 6H

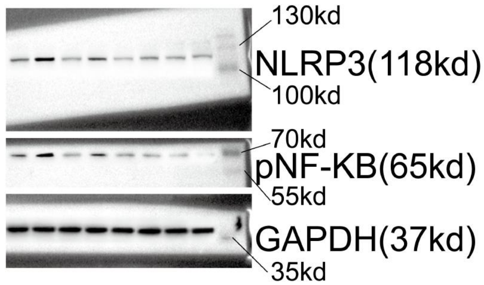


**Supplemental Figure S7.** Western blot results with molecular weight

DOI [to be added]
